# Per- and Polyfluoroalkyl Substances (PFAS) Accumulation, Reproductive Impairment, and Associations with Nestling Body Condition in Great (*Parus major*)- and Blue Tits (*Cyanistes caeruleus*) Living near a Hotspot in Belgium

**DOI:** 10.3390/toxics12090636

**Published:** 2024-08-29

**Authors:** Thimo Groffen, Jodie Buytaert, Els Prinsen, Lieven Bervoets, Marcel Eens

**Affiliations:** 1 ECOSPHERE, Department of Biology, Faculty of Science, University of Antwerp, Groenenborgerlaan 171, 2020 Antwerp, Belgium; jodie.buytaert@uantwerpen.be (J.B.); lieven.bervoets@uantwerpen.be (L.B.); 2 Behavioural Ecology and Ecophysiology Group, Department of Biology, Faculty of Science, University of Antwerp, Universiteitsplein 1, 2610 Wilrijk, Belgium; marcel.eens@uantwerpen.be; 3 Integrated Molecular Plant Physiology Research, Department of Biology, Faculty of Science, University of Antwerp, Groenenborgerlaan 171, 2020 Antwerp, Belgium; els.prinsen@uantwerpen.be

**Keywords:** perfluoroalkyl substances, birds, eggs, plasma, reproductive impairment

## Abstract

Due to the limited number of field studies investigating associations between environmentally relevant per- and polyfluoroalkyl substances (PFAS) mixtures and reproductive impairment, there is uncertainty as to whether birds are affected by PFAS pollution, whether species differ in sensitivity to PFAS, and whether the observed reproductive impairment is caused by PFAS or rather due to other potential confounding variables. Therefore, we investigated PFAS concentrations in eggs and blood plasma of great tit (*Parus major*) and blue tit (*Cyanistes caeruleus*) nestlings near a PFAS hotspot in Belgium, reproductive impairment, and associations between the accumulated levels and nestling body condition. In total, 29 eggs and 22 blood plasma samples of great tit clutches, and 10 egg and 10 blood plasma samples of blue tit clutches, were collected. Despite more types of PFAS being detected in eggs compared to plasma, only minor differences in profiles were observed between species. On the other hand, tissue-specific differences were more pronounced and likely reflect a combination of maternal transfer and dietary exposure post-hatching. Despite the high concentrations detected in both species, limited reproductive impairment was observed. Our results support previous findings that great tits and blue tits may not be very susceptible to PFAS pollution and provide evidence that other factors, including ecological stoichiometry, may be more important in explaining inter-species variation in PFAS accumulation and reproductive impairment.

## 1. Introduction

Per- and polyfluoroalkyl substances (PFAS) are a large group of synthetic organic chemicals that consist of at least one fully fluorinated methyl or methylene carbon atom (without any H/Cl/Br/I atom attached to it) [1]. Their chemical structure results in typical properties (e.g., thermal and chemical stability, lipophobicity, and hydrophobicity), which makes them useful in numerous applications [2]. Many PFAS are highly persistent and mobile, which, combined with their widespread use in industrial processes and consumer products, has resulted in their global distribution in the environment and in biota [3,4,5,6]. PFAS present in the environment may bioaccumulate in organisms and some PFAS are known to biomagnify in food chains [7,8,9].

Birds are particularly susceptible to environmental pollution due to their relatively high trophic level and efficient respiratory system [10]. The majority of avian ecotoxicological studies on PFAS have been conducted in the laboratory, using model species such as chickens (*Gallus* spp.), quails (e.g., *Colinus* sp., *Coturnix* sp.), and ducks (*Anas* sp.) (reviewed by Ankley et al. [11]). Although these studies predict whether, and at what dose, PFAS are likely to affect populations of these species, the outcomes may not necessarily reflect those of wild populations [11,12]. Despite the greater variety in the response of organisms to environmental stressors in field studies, caused by potential confounding factors, field studies reflect effects on organisms in their native environment and are thus necessary for ecological risk assessment purposes.

The reproductive effects of PFAS have been studied in various bird species [11,12]. The review by Ankley et al. [11] reported that many laboratory studies have only focused on perfluorooctane sulfonate (PFOS) as it is usually the PFAS present in the highest concentration in the environment. Since wild birds are exposed to varying PFAS mixtures, which can have additive, synergistic, or even antagonistic effects, it is important to investigate whether these mixtures may cause reproductive impairment (i.e., any interference with the ability to reproduce) or whether other PFAS than PFOS may be more relevant [13,14]. Field studies on reproductive impairment in tree swallows (*Tachycineta bicolor*) and great tits (*Parus major*) revealed that species may differ in sensitivity to PFAS pollution [13,14,15,16,17], although it has been hypothesized that other factors, such as differences in composition of environmental PFAS mixtures among sites, presence of co-contaminants, differences in available resources, etc., may be more important in explaining this variation [12]. Nonetheless, due to the limited number of field studies investigating the associations between environmentally relevant PFAS mixtures and reproductive impairment in birds, more research is needed to investigate whether bird species differ in sensitivity and to confirm whether previously reported associations with reproductive impairment are likely caused by PFAS pollution or rather an effect of ecological stoichiometry (i.e., effects of an imbalance between available resources (e.g., food) and the physiology of an organism (e.g., the physiology of reproduction [12]) and other confounding variables [12].

To meet this end, known hotspot areas such as fluorochemical plants provide the ideal study sites as toxic effects in laboratory studies have been observed at concentrations that are mainly found at these sites [8,18]. Therefore, the objective of this study was to determine the concentrations of 29 PFAS, including both legacy and emerging PFAS, in the eggs and blood plasma of great tits (*Parus major*) and blue tits (*Cyanistes caeruleus*) near a hotspot in Zwijndrecht (Belgium), to investigate reproductive toxicity, and to associate the accumulated concentrations with nestling body condition in both species. Furthermore, we investigated the species-specific differences and tissue-specific differences in accumulation profiles and concentrations.

High PFAS concentrations in the eggs and blood plasma of the nestlings were expected since this hotspot in Belgium is known to contain among the highest PFAS concentrations in the world [14,19,20,21]. We hypothesize that PFAS are detected in high concentrations and that more PFAS can be detected in the blood plasma, as this matrix represents exposure through both maternal transfer and dietary sources, whereas egg concentrations only represent maternal transfer. Blue tits are expected to have lower PFAS concentrations and a less diverse PFAS profile compared to great tits. The lifespan of blue tits is shorter than great tits, and thus have less exposure over time; blue tits forage arboreally only, whereas great tits forage both arboreally and on the ground [22]. A previous study reported that great tits may not be very susceptible, in terms of reproductive toxicity, to PFAS pollution [14]. Thus, we expect very similar results, with limited reproductive impairment in great tits. In addition, since blue tits also breed in contaminated areas [22], we do not expect large species-specific differences in sensitivity to PFAS pollution.

## 2. Materials and Methods

### 2.1. Study Species and Sample Collection

**Figure 1:**
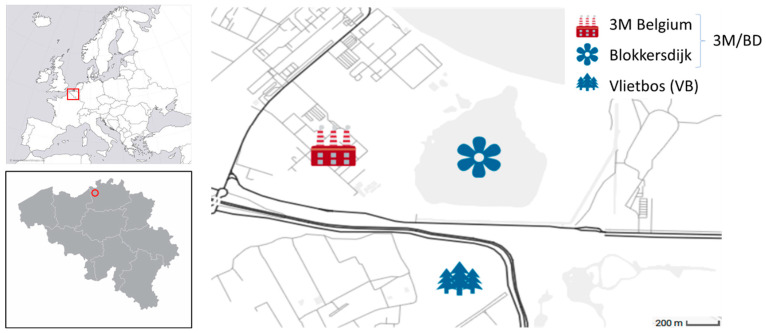
Overview of the study area in Antwerp, Belgium. 3M (latitude, longitude: 51.233389, 4.334882) and Blokkersdijk (51.232236, 4.347188) were considered one sampling site (‘3M/BD’) due to overlapping foraging and nesting areas. Vlietbos (VB; 51.223875, 4.347621) was considered a separate sampling site. The red frame and circle show the positioning of the sampling site within Belgium and Europe, respectively.

During autumn of 2021, nest boxes were installed at the 3M site in Zwijndrecht (Antwerp region, Belgium), the neighboring nature reserve Blokkersdijk (BD), and at Vlietbos (VB), a forest area approximately 1.5 km from 3M (Figure 1). Nest boxes that were still present from previous studies in 2016 were either cleaned or replaced. The great and blue tit populations at these sites are well established as nest boxes have been present in these areas for numerous years [19,20,23]. The 3M and Blokkersdijk sites were taken together and considered as one site in this study, due to overlapping foraging and nesting areas. In total, 38 and 19 nest boxes were installed at 3M/BD and VB, respectively.

From well before the presumed start of egg laying (breeding season 2022; late February–mid-June) until incubation, nest boxes were checked daily to be able to determine the start of the egg-laying period (i.e., egg-laying day, with day 0 being the day on which the first egg was laid in any of the sampling sites) and clutch size (i.e., total amount of eggs in a clutch when incubation had started). From each nest, a random egg was collected by hand, when 3–4 eggs were present in the clutch, before incubation had started. The eggs were stored in polypropylene (PP) tubes at −20 °C prior to PFAS analysis. From 10 days after incubation (the absence of coverage of the eggs was used as an indicator for the start of incubation) onwards, the nests were checked daily for hatching to determine hatching success (i.e., number of hatched eggs divided by the number of incubated eggs, including nests where no eggs hatched). Body condition of the nestlings was determined 14 days post-hatching according to the scaled mass index of Peig and Green [24]. Briefly, a standardized major axis regression on ln-transformed data of body mass and tarsus length was performed to determine the slope of the model, after which the scaled mass index ${\hat{M}}_{i}$ was computed following equation 1, with M_i_ and L_i_ the body mass and tarsus length of individual *i*, *b_SMA_* the scaling exponent calculated by dividing the slope of the ordinary least squares regression by Pearson’s correlation coefficient, and *L*_0_ the arithmetic mean of the study population [24].

(1) $${\hat{M}}_{i}=M_{i}\;{\left[\frac{L_{0}}{L_{1}}\right]}^{b_{SMA}}$$

At the same time, a blood sample was taken from the brachial vein of the nestlings, and a maximum of 150 μL (pooled per clutch) was collected in microhematocrit heparinized capillary tubes (Microvette CB 300). These samples were centrifuged at 3000 rpm for 10 min in a microcentrifuge to separate red blood cells from the plasma. The plasma was transferred into PP Eppendorf tubes and stored at −80 °C prior to PFAS analysis. Finally, the nest boxes were checked approximately 25 days post-hatching to determine fledging success (i.e., number of fledglings divided by the number of hatched eggs). In total, 29 great tits (N = 16 at 3M/BD, N = 13 at VB), and 10 blue tits (N = 6 at 3M/BD, N = 4 at VB) eggs were collected. Blood plasma of 22 clutches of great tits (N = 14 at 3M/BD, N = 8 at VB) and 10 clutches of blue tits (N = 6 at 3M/BD, N = 4 at VB) were sampled. The nest boxes were specifically adapted to great tits (bigger opening, resulting in competition between blue and great tit), explaining the smaller sample sizes for blue tits. Eggshells were removed prior to PFAS analysis and the thickness of the eggshells was measured, after removal of the inner membrane, in three small pieces (approximately 0.5 × 0.5 cm) from the equatorial region using a micrometer (±0.01 mm, Mitutoyo Belgium NV, Kruibeke, Belgium), following the protocol described by Lopez-Antia et al. [25]. The average thickness was calculated based on the three measurements.

### 2.2. PFAS Extraction and Analysis

Whole egg content was homogenized by repeatedly sonicating and vortex-mixing. Approximately 200 mg of homogenized egg and 20 μL of plasma were used for the analysis. The extraction procedure for eggs was described and validated by Powley et al. [26], whereas the method for the plasma followed the procedure of Groffen et al. [27].

Each sample was spiked with 10 ng of a heavy-labeled perfluoroalkyl carboxylic acid (PFCA) and perfluoroalkyl sulfonic acid (PFSA) mixture (MPFAC-MXA, Wellington Laboratories, Guelph, ON, Canada). After adding 10 mL of acetonitrile (ACN; HPLC gradient grade, Acros Organics BVBA, Geel, Belgium), the samples were vortex-mixed and sonicated (Branson 2510, VWR International, Leuven, Belgium) for 3 × 10 min, with vortex-mixing in between. Hereafter, all samples were placed overnight on a shaking plate (GFL 3020, VWR International, Leuven, Belgium) at 135 rpm and room temperature. After vortex-mixing, the samples were centrifuged (4 °C, 1037× *g*, 10 min; Eppendorf centrifuge 5804R, Eppendorf Belgium N.V.-S.A., Aarschot, Belgium) and the supernatant was transferred into new PP tubes. The egg extracts were dried under vacuum to approximately 0.5 mL using a rotational-vacuum-centrifuge (Eppendorf concentrator 5301, Hamburg, Germany) and then transferred to PP Eppendorf tubes containing 0.1 mL of graphitized carbon powder (Supelclean ENVI-Carb, Sigma-Aldrich, Overijse, Belgium) and 50 μL of glacial acetic acid (Fisher Scientific, Merelbeke, Belgium). The empty PP tubes, that used to contain the extracts, were rinsed twice with 250 μL of ACN, which was also added to the Eppendorf tube. After vortex-mixing for at least 1 min, the samples were centrifuged (4 °C, 9279.4× *g*, 10 min; Eppendorf centrifuge 5415R, Eppendorf Belgium N.V.-S.A., Belgium) and the supernatant was dried completely using the vacuum-centrifuge.

The supernatants of the plasma samples were loaded onto Chromabond HR-XAW SPE cartridges (Macherey-Nagel, Düren, Germany) that were preconditioned using 5 mL of ACN and 5 mL of Milli-Q water. After loading, the cartridges were washed with 5 mL of a 25 mM ammonium acetate solution (dissolved in Milli-Q) and 2 mL of ACN. Finally, the PFAS were eluted using 2 × 1 mL of a 2% ammonium hydroxide solution (Thermo Scientific, Belgium; diluted in ACN). The eluent was dried completely under vacuum.

Finally, all samples were reconstituted with 200 μL of a 2% ammonium hydroxide solution (diluted in ACN), vortex-mixed, and filtered through an Ion Chromatography Acrodisc 13 mm syringe filter with 0.2 μm Supor polyethersulfone (PES) membrane (VWR International, Belgium) into a PP auto-injector vial. Procedural blanks (10 mL of ACN) for both matrices followed the same protocols.

The samples were analyzed using ultra-performance liquid chromatography–tandem mass spectrometry (UPLC-MS/MS, ACQUITY TQD, Waters, Milford, MA, USA) using negative electrospray ionization. To separate the target analytes, an ACQUITY BEH C18 column (2.1 × 50 mm; 1.7 μm, Waters, Milford, MA, USA) was used. In addition, an ACQUITY BEH C18 pre-column (2.1 × 30 mm; 1.7 μm, Waters, Milford, MA, USA) was inserted between the solvent mixer and injector to retain any PFAS contamination originating from the system. As mobile phase solvents, a 0.1% formic acid in water solution (HPLC grade, VWR International, Belgium) and a 0.1% formic acid (LC/MS grade, Fisher Chemical, Merelbeke, Belgium) in ACN solution were used. The injection volume was set at 6 μL and the flow rate was 450 μL/min. The solvent gradient started at 65% of 0.1% formic acid in water, went to 0% in 3.4 min, and back to 65% at 4.7 min. Multiple reaction monitoring (MRM) of two diagnostic transitions per PFAS analyte or internal standard was used to identify and quantify the 29 targeted legacy and emerging PFAS (Tables S1 and S2). The diagnostic transitions were validated by Groffen et al. [27,28].

### 2.3. Quality Control and Assurance

One procedural blank, consisting of 10 mL of ACN, was included per batch of 15 samples to detect any contamination that may have occurred during the extraction and analysis. Any contamination in these blanks was subtracted from the concentrations in the samples within the same batch. As instrumental blanks, ACN was regularly injected to prevent cross-over contamination between injections. The limits of quantification (LOQ) of each analyte were determined in the matrix as the concentration corresponding to a signal-to-noise ratio of 10 and are shown in Table S1 for eggs and Table S2 for plasma.

### 2.4. Statistical Analysis

The statistical analyses were performed using R Studio (version 2023.12.1 + 402; R version 4.2.2). The Shapiro–Wilk test was used to examine the validity of the models’ assumptions, and data were log-transformed when needed to fulfill the normality assumptions. The levels of significance were set a *p* ≤ 0.05. Concentrations below the LOQ were substituted following a maximum likelihood estimation method [29].

The PFAS composition profiles were calculated as the proportions of individual compounds to the total PFAS concentration, taking the molecular weight of each individual PFAS into account. These percentages were then averaged per site and species for both eggs and plasma. Differences in PFAS concentrations between sites and species were examined using a two-way ANOVA, followed by Tukey’s honest significant differences post hoc analysis. Correlations between egg and plasma concentrations were assessed using Spearman rank correlation analyses. Two-sample *t*-tests (or Wilcoxon rank sum tests in case of non-normality) were used to compare the reproductive parameters (i.e., egg-laying day, shell thickness, clutch size, hatching success, and fledging success) and body condition between the sites.

To account for collinearity, a principal component analysis (PCA) was conducted on the detected PFAS in both plasma and eggs, for both great tits and blue tits separately. PFAS that were correlated within the same matrix and for the same species, were grouped together and given a replacement value that was determined by taking the average concentration of all PFAS within this group. This resulted in four subgroups for great tit eggs (i.e., (1) PFBS, (2) PFDS, PFUnDA, PFDoDA, PFTrDA, PFTeDA, (3) PFNA, PFDA, PFOS, (4) PFHxA, PFOA, PFHxS, PFHpS), three subgroups for great tit plasma ((1) PFBS, PFEESA, (2) PFOA, PFOS, PFNA, (3) PFBA, PFDoDA, PFTrDA), four subgroups for blue tit eggs ((1) PFBS, (2) PFTeDA, (3), PFDS, PFUnDA, PFDoDA, PFTrDA, (4) PFHxS, PFHpS, PFOS, PFOA, PFNA, PFDA), and two subgroups for the blue tit plasma ((1) PFBA, PFOA, PFOS, (2) PFBS, PFEESA). Generalized linear models were then used to correlate the concentrations of these subgroups to the reproductive parameters and body condition of the nestlings, using the following distributions: a Poisson distribution was used to study correlations with clutch size, a normal distribution to study correlations with the egg-laying date, eggshell thickness, and the mean condition of the nestlings, and a binary logistic distribution for hatching success (number of hatched eggs divided by the number of incubated eggs) and fledging success (number of fledglings divided by the number of hatched eggs).

## 3. Results

### 3.1. PFAS Concentrations and Accumulation Profiles

**Figure 2:**
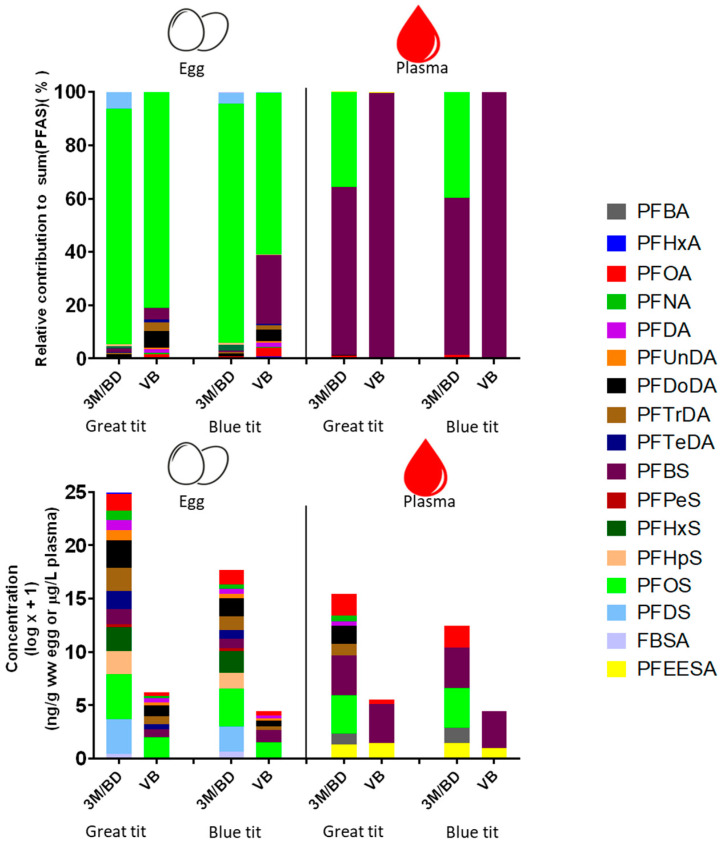
PFAS profiles (%) and concentrations (log x + 1 transformed) in eggs (ng/g ww) and blood plasma (pooled per clutch) of chicks (μg/L) from 3M and Blokkersdijk (3M/BD; N = 16 great tit eggs, N = 6 blue tit eggs, N = 14 great tit plasma, N = 6 blue tit plasma), and Vlietbos (VB; N = 13 great tit eggs, N = 4 blue tit eggs, N = 8 great tit plasma, N = 4 blue tit plasma). Detailed information about the PFAS analytes and their concentrations in eggs and plasma can be found in Table S1 and Table S2, respectively.

Out of the 29 PFAS targeted (Tables S1 and S2), 17 PFAS were detected in the egg samples and 9 in the plasma samples. Substantial differences between sampling sites can be observed in terms of both absolute PFAS concentrations (Figure 2; Tables S1 and S2) and the relative contribution of each PFAS to the total PFAS burden (Figure 2). Concentrations of PFHxA, PFOA, PFNA, PFDA, PFUnDA, PFDoDA, PFTrDA, PFTeDA, PFBS, PFHxS, PFHpS, PFOS, and PFDS in great tit eggs were significantly higher at 3M/BD than at VB (*p* < 0.05). The same pattern was observed for concentrations of PFOA, PFDoDA, PFTrDA, PFHxS, PFHpS, PFOS, and PFDS in blue tit eggs (*p* < 0.05). In plasma, the concentrations of PFOS and PFOA were higher at 3M/BD than at VB for both species (*p* < 0.05), while no difference was observed between sites for PFEESA and PFBS (*p* > 0.05). The detection of specific PFAS differed primarily between sites. For example, PFHxA, PFPeS, PFHxS, PFHpS, PFDS, and FBSA were detected in eggs collected at 3M/BD, but not in eggs from VB (the site further away from 3M). Similarly, PFBA, PFNA, PFDA, PFDoDA, PFTrDA, and PFOS in blood plasma were also only detected at 3M/BD.

The PFAS concentrations in the eggs and plasma did not differ significantly between species (*p* > 0.05). Although PFAS accumulation profiles between species were very similar, the profile of blood plasma collected from great tits at 3M/BD contained more different types of PFAS than that of blue tits from the same site (Figure 2). This difference was less pronounced in the eggs. Finally, differences between both matrices were observed, with PFBS being dominant in the plasma and PFOS in the eggs (Figure 2). PFBA and PFEESA were the only two PFAS that were detected in plasma but were <LOQ in the eggs, whereas PFHxA, PFUnDA, PFTeDA, PFPeS, PFHxS, PFHpS, PFDS, and FBSA showed the opposite pattern.

### 3.2. Correlations between Egg and Plasma Concentrations

**Figure 3:**
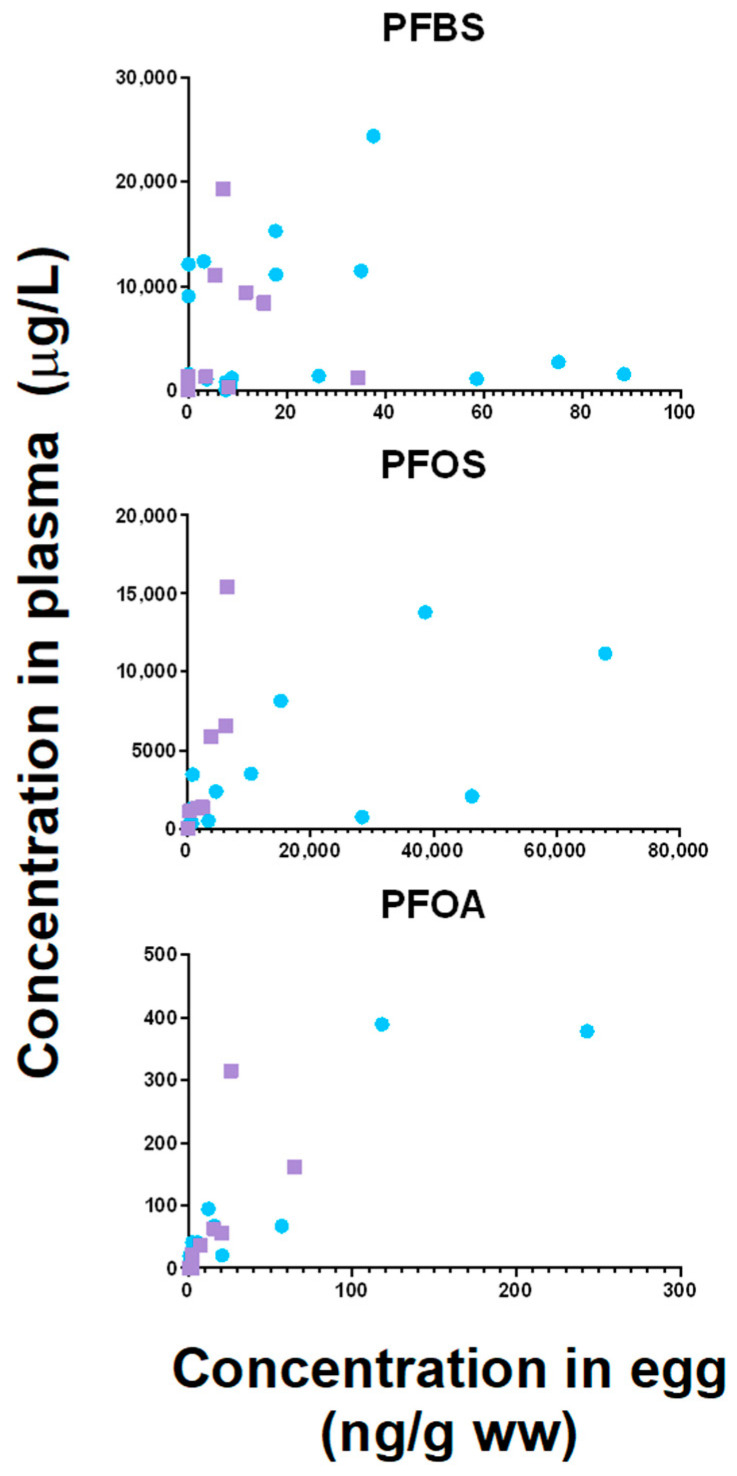
Correlations between plasma and egg PFAS concentrations. Only PFAS with >30% detection in both matrices were used for studying associations between PFAS concentrations in plasma and eggs. PFOS and PFOA concentrations were, for both species, significantly correlated between both matrices (*p* < 0.05).

Since only PFBS, PFOA, and PFOS were detected in more than 30% of both egg and plasma samples, only these three PFAS were correlated between both matrices. Concentrations of PFOA (ρ = 0.642, *p* = 0.002 for great tit; ρ = 0.855, *p* = 0.003 for blue tit) and PFOS (ρ = 0.853, *p* < 0.001 for great tit; ρ = 0.879, *p* = 0.002 for blue tit) were both positively correlated between eggs and plasma of both species (Figure 3), whereas no correlation was observed for PFBS (Figure 3).

### 3.3. Reproductive Toxicity and Associations with Nestling Body Condition

**Figure 4:**
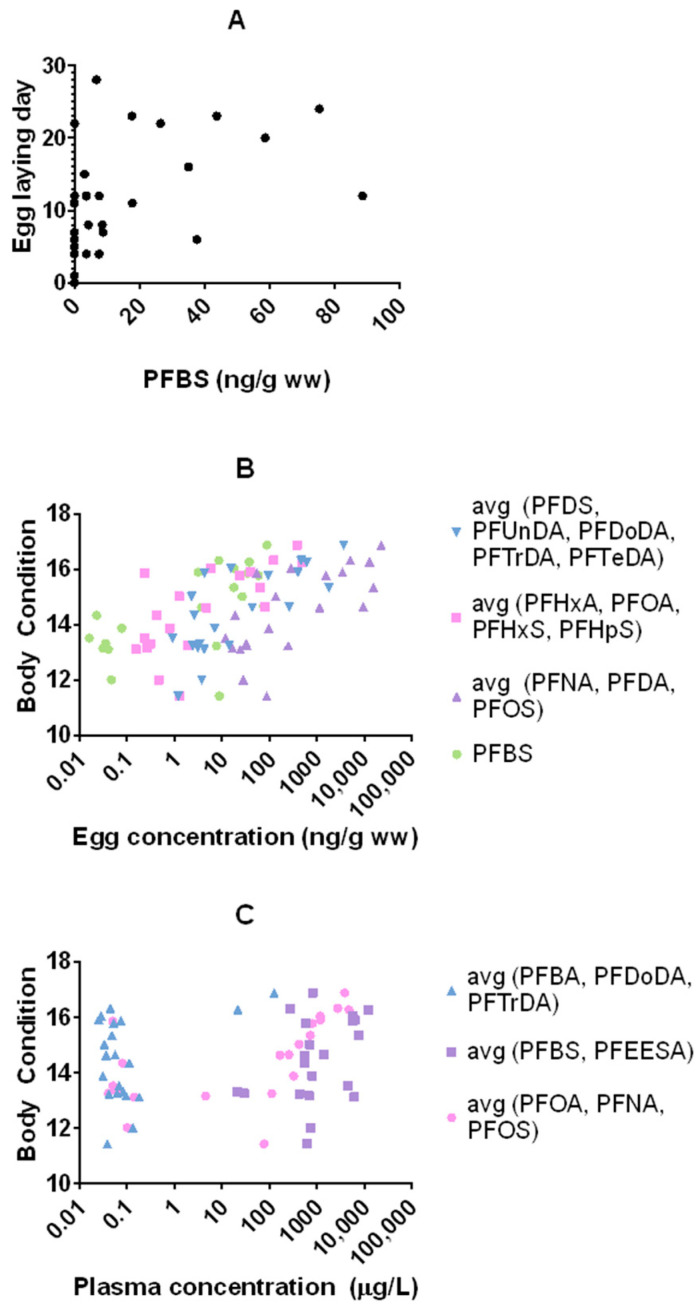
Associations between (**A**) PFBS in egg of great tit and day of the first egg (day 0 is the day the first egg was laid), (**B**) body condition of great tit nestlings and egg PFAS concentrations (the *x*-axis is on log-scale), (**C**) body condition of great tit nestlings and plasma PFAS concentrations (the *x*-axis is on log-scale). Principal component analysis revealed collinearity among PFAS. Therefore, some PFAS were grouped (by taking the average (avg) of the PFAS within that group) prior to the generalized linear models.

Reproductive parameters (i.e., egg-laying day, shell thickness, clutch size, hatching success, and fledging success) and nestling body condition per species and site are shown in Table S3. Both species started laying earlier at Vlietbos (*p* < 0.05) and nestlings of both species had a higher average body condition at 3M/BD (*p* < 0.05). In addition, great tit eggs had thinner eggshells at 3M/BD compared to Vlietbos (*p* = 0.002), and there were two trends observed with great tit nests having a higher hatching and fledging success at 3M/BD compared to VB (*p* = 0.061 and *p* = 0.083, respectively). Clutch sizes did not significantly differ between sites for both species (*p* > 0.05). The generalized linear models on the grouped PFAS variables (see 2.4) revealed that PFBS concentrations in the eggs of great tits were positively associated (*p* = 0.013) with the egg-laying date (Figure 4A). Furthermore, significant positive associations between nestling body condition and great tit egg concentrations of (1) PFBS (*p* = 0.007); (2) the average of PFDS, PFUnDA, PFDoDA, PFTrDA, and PFTeDA (*p* = 0.022); (3) the average of PFNA, PFDA, and PFOS (*p* = 0.007); and (4) the average of PFHxA, PFOA, PFHxS, and PFHpS (*p* = 0.010) were observed (Figure 4B). Great tit plasma concentrations of the average of PFOA, PFOS, and PFNA were also positively associated (*p* < 0.001) with nestling body condition, whereas concentrations of the average of PFBS and PFEESA (*p* = 0.080), and the average of PFBA, PFDoDA, and PFTrDA (*p* = 0.069) showed trends (Figure 4C). No associations between PFAS concentrations and clutch size, hatching and fledging success, and shell thickness were observed for great tits (*p* > 0.05). Accumulated concentrations in blue tits were not associated with any of the investigated parameters (*p* > 0.05).

## 4. Discussion

### 4.1. PFAS Accumulation: Tissue- and Species-Specific Differences

The majority of studies examining wildlife exposure to PFAS provide snapshots of PFAS levels in single tissue compartments, hence limiting the assessment of the total exposure across multiple tissues and organs [30,31]. Studies examining PFAS accumulation in multiple tissues of wild birds have revealed the partitioning behavior of PFAS [30,32,33,34,35]. These tissue-specific measurements indicated interactions with specific proteins such as albumin or liver fatty acid binding protein (L-FABP), which determine PFAS partitioning in matrices like blood and plasma [36].

Although PFAS concentrations in birds tend to differ between eggs and plasma, the same PFAS are often detected in both matrices, albeit with different contributions to the sum of accumulated PFAS in these matrices [14,21,32,34]. Despite this being contradictory to our findings, i.e., more types of PFAS were detected in the eggs than in the plasma, the differences observed in the present study could be the result of a lower detection sensitivity, hence higher LOQ values, for some PFAS in plasma.

The similarities in PFAS profiles between blood plasma and eggs suggest maternal transfer of some PFAS, which confirms earlier findings in birds [21,34,37,38]. Even though we did correlate the concentration in one egg, which might not be representative of the entire clutch [39], with an average concentration in the nestling plasma, the positive associations between egg and plasma concentrations of PFOA and PFOS in the present study further support the hypothesis of maternal transfer.

On the other hand, the dissimilarities between both matrices suggest that other sources (e.g., dietary sources) could play a more prominent role in the accumulation profiles in plasma (e.g., in the case of PFBA and PFEESA, which were only detected in the plasma). Another explanation that requires further examination, is that some PFAS present in eggs may accumulate in tissues other than blood plasma in developing nestlings. Compound-specific differences in affinity for various organs and tissues have been reported in juvenile seabirds [30]. Even though the accumulation profiles and concentrations did not differ much between both species, the minor differences that were observed could be explained by differences in diet and foraging habits of both species, which has also been suggested by Lasters et al. [22].

### 4.2. Temporal Trends

Previous studies on passerine birds near this hotspot reported sharply decreasing concentrations of some PFAS with the distance from the 3M site [14,19,21], something which was also observed in the present study. In addition, these avian monitoring studies in this area have reported among the highest concentrations ever observed in eggs and plasma of various bird species. Although fewer types of PFAS were targeted in these previous studies, and other sampling sites have sometimes been included in the reported concentration ranges, a comparison of PFAS concentrations revealed that the PFOS concentrations in blue tit eggs in the present study (14.0–6483 ng/g ww) were comparable to those collected in 2016 (<11 to 6743 ng/g ww [22]), whereas those of PFOA (<0.04 to 359 ng/g ww [22]) were more than five times higher in 2016 compared to the present study.

A comparison of great tit egg concentrations at 3M and Vlietbos between 2011 [19], 2016 [14], and 2022 (the present study) not only showed that, when comparing the same number of targeted analytes, more types of PFAS were detected in the present study compared to previous years, but also that temporal trends differ depending on the type of PFAS (Table 1). For example, concentrations of PFBS, PFDS, and the long-chain carboxylic acids (PFUnDA, PFDoDA, PFTrDA, and PFTeDA) have increased compared to previous years, whereas those of PFOS and PFDA have decreased substantially compared to 2016, and those of PFOA and PFNA have remained stable. Plasma concentrations of PFBA, PFOS, PFUnDA, and PFTeDA have decreased compared to 2016 [21], whereas those of PFOA, PFNA, and PFDA remained similar. A substantial increase in plasma concentrations over time was observed for PFBS, PFDoDA, and PFTrDA.

Despite the phase-out of PFOS by 3M in the early 2000s [40] the PFOS concentrations had increased between 2011 and 2016, which Groffen et al. [14] hypothesized to be due to a lagged ecosystem response to atmospheric concentrations (including those of precursor compounds). More specifically, since changes in their chemical production are hypothesized to first affect atmospheric concentrations, PFAS and precursors that were already present in the atmosphere could still end up in the environment through deposition, which could explain why concentrations remained stable or even increased after regulations [14]. On the other hand, the absence of any temporal trend could also be related to the persistence of these PFAS in the environment (e.g., the estimated half-life for PFOA in the environment was reported to be >92 years [41]). The present study suggests that environmental PFOS concentrations have started to decrease and that the effects of the industrial phase-out may become more visible. On the other hand, the shift in production from PFOS to PFBS [42] might explain why PFBS concentrations and relative contributions increased over time. The increased concentrations of long-chain carboxylic acids were consistent with findings in chicken eggs in the vicinity of the same fluorochemical plant and were hypothesized to be caused by atmospheric oxidation of precursors such as fluorotelomer alcohols [43]. Concerning the PFAS that were present in lower concentrations (i.e., close to LOQ values such as PFHxS and other short-chained carboxylic acids), temporal trends could have been influenced by differences in analytical sensitivity for these compounds over time. Overall, temporal trends have been shown to be highly variable among studies and types of PFAS, with studies reporting either insignificant trends, increases, or decreases over time [43,44,45,46,47]. These differences are likely due to site-specific differences in environmental conditions and PFAS emissions, and species-specific differences in ecology [45]. Investigating the presence of PFAS precursors in the atmosphere and environment would provide more insights into how PFAS concentrations respond to regulations and phase-outs.

### 4.3. Reproductive Toxicity and Associations with Nestling Body Condition

The absence of indications of reproductive toxicity confirms earlier findings that great tits may not be very susceptible to PFAS pollution [14] and shows that this might also be true for blue tits, although the results of blue tits should be treated with caution due to the smaller sample size (N = 6 at 3M/BD and N = 4 at VB, for both eggs and plasma).

Exposure to PFDA was associated with a reduced hatching success and total breeding success in great tits [14], something which was not observed in the present study. In addition, PFOS exposure caused embryo death and a reduced hatching success in tree swallows from Minnesota and Wisconsin, USA [15,16]. On the other hand, no demonstrable effects of PFAS exposure on tree swallow reproduction were reported in northeastern Michigan, USA [17].

Delayed laying is typically associated with poor reproductive output [48]. Although our results suggest that PFBS may be associated with delayed laying in great tits, we did not observe any other associations with reproductive output, indicating that other factors may have more influence on laying delay. The PFAS concentrations (including PFBS) in the present study were particularly high at 3M/BD, meaning that the observed delay in laying could be related to differences in stoichiometry between sites rather than an effect of PFBS. However, since food availability was not investigated, this hypothesis needs to be examined in future studies.

The positive associations between most PFAS and the body condition of the nestlings show that clutches with, on average, larger nestlings have higher accumulation of PFAS compared to clutches with smaller nestlings. Nestlings with a better condition (i.e., heavier or bigger nestlings) are typically those that are fed more frequently or are fed with higher quality food sources [49] and these associations could thus be the result of food availability and quality rather than PFAS exposure. In addition, inter-clutch competition for food, where bigger nestlings receive more food and may thus be exposed more to PFAS from dietary sources [50], could potentially affect the observed associations. Nonetheless, this requires confirmation by future studies in which PFAS concentrations of the individual nestlings are related to their body condition.

Overall, our findings support the hypotheses by Custer [12] that other factors, including ecological stoichiometry, may be more important in explaining inter-species variation in associations between accumulated PFAS concentrations and reproductive impairment.

## Figures and Tables

**Table 1:** Mean concentrations and ranges of PFAS concentrations (ng/g ww) in great tit eggs collected at 3M and Vlietbos in previous studies and the present study in the vicinity of the fluorochemical plant in Zwijndrecht. Only PFAS that were targeted in all three studies have been included. The results of 2011 and 2016 only include those of the 3M site since Blokkersdijk was not included as a study area in those studies. No range is given if all concentrations were <LOQ. ND = not detected.

	2011 Groffen et al. [19]		2016 Groffen et al. [14]		2022 (The Present Study)	
	3M	Vlietbos	3M	Vlietbos	3M/BD	Vlietbos
PFBS	ND	ND	ND	ND	24.9 (<0.762–88.5)	4.45 (<0.762–35.1)
PFHxS	162 (36.9–355)	1.6 (<0.45–5.6)	ND	ND	166 (<2.79–1005)	<2.79
PFOS	20,122 (3237–69,218)	254 (55.1–782)	48,056 (5111–187,032)	830 (<2.55–4035)	15,545 (261–67,916)	101 (34.5–253)
PFDS	ND	ND	315 (9.4–1489)	<5.92	1939 (1.61–13,765)	<0.587
PFBA	ND	ND	1.7 (<0.261–11)	<0.261 (<0.261–1.7)	ND	ND
PFPeA	ND	ND	ND	ND	ND	ND
PFHxA	ND	ND	ND	ND	0.431 (<0.375–1.25)	<0.375 (<0.375–0.440)
PFHpA	ND	ND	ND	ND	<0.485 (<0.485–4.03)	<0.485
PFOA	26.9 (2.7–56.3)	1.0 (0.3–1.9)	39 (3.4–359)	1.8 (<0.045–3.5)	33.3 (0.836–243)	1.12 (0.503–2.22)
PFNA	4.2 (<1.8–20.5)	<1.8	9.1 (2.1–28)	1.8 (<0.586–5.7)	7.13 (<0.198–26.4)	0.565 (<0.198–1.48)
PFDA	12.3 (<1.4–37.2)	<1.4	25 (1.6–102)	0.7 (<0.425–4.1)	7.71 (<0.334–26.9)	1.47 (0.641–2.67)
PFUnDA	ND	ND	ND	ND	8.60 (<0.230–53.7)	0.995 (0.344–2.17)
PFDoDA	22.0 (2.0–104)	0.7 (<0.32–1.50)	29 (1.1–133)	1.8 (<0.444–7.8)	355 (3.04–2953)	8.94 (2.61–21.5)
PFTrDA	7.9 (<0.38–32.3)	0.4 (<0.38–0.9)	25 (<0.256–156)	6.0 (<0.256–22)	139 (1.19–1034)	5.07 (1.46–13.9)
PFTeDA	ND	ND	3.4 (<0.355–22)	1.2 (<0.355–4.1)	50.1 (<0.535–450)	1.77 (<0.535–5.10)

## Data Availability

The original contributions presented in the study are included in the article/Supplementary Material, while further inquiries can be directed to the corresponding author.

## Supplementary Materials

The following supporting information can be downloaded at: https://www.mdpi.com/article/10.3390/toxics12090636/s1, Table S1: PFAS concentrations and limit of quantification (LOQ) in egg (ng/g ww) of great tit and blue tit. Values represent average and range (min–max, between brackets). Sample sites represent the combination of 3M and Blokkersdijk (3M/BD) and Vlietbos (VB). Great tit eggs: N = 16 and N = 13 for 3M/BD and VB, respectively. Blue tit eggs: N = 6 at 3M/BD and N = 4 at VB. Average concentrations below the LOQ are shown as ‘<LOQ’. No range is given if all concentrations were <LOQ.; Table S2: PFAS concentrations and limit of quantification (LOQ) in plasma (μg/L) of great tit and blue tit. Values represent average and range (min–max, between brackets). Sample sites represent the combination of 3M and Blokkersdijk (3M/BD) and Vlietbos (VB). Great tit plasma: N = 14 at 3M/BD, N = 8 at VB. Blue tit plasma: N = 6 at 3M/BD, N = 4 at VB. Average concentrations below the LOQ are shown as ‘<LOQ’. No range is given if all concentrations were <LOQ.; Table S3: Mean values and standard error for the different reproductive parameters and nestling body condition at the different locations for both great tit and blue tit.
