# Supplementary material for: Per- and Polyfluoroalkyl Substances (PFAS) Accumulation, Reproductive Impairment, and Associations with Nestling Body Condition in Great (Parus major)- and Blue Tits (Cyanistes caeruleus) Living near a Hotspot in Belgium
Source: Toxics. 2024 Aug 29;12(9):636. doi: 10.3390/toxics12090636 (PMC11435652; doi:10.3390/toxics12090636)
Supplement: Supplementary file 1 [file toxics-12-00636-s001.zip › toxics-3141302-supplementary.pdf]

toxics

Supplementary Materials: Per- and Polyfluoroalkyl Substances (PFAS) Accumulation, Reproductive Impairment, and Associations with Nestling Body Condition in Great (Parus major)- and Blue Tits (Cyanistes caeruleus) Living near a Hotspot in Belgium

Thimo Groffen, Jodie Buytaert, Els Prinsen, Lieven Bervoets and Marcel Eens

Table S1. PFAS concentrations and limit of quantification (LOQ) in egg (ng/g ww) of great tit and blue tit. Values represent averages and range (min – max, between brackets). Sample sites represent the combination of 3M and Blokkersdijk (3M/BD) and Vlietbos (VB). Great tit eggs: N = 16 and N = 13 for 3M/BD and VB, respectively. Blue tit eggs: N = 6 at 3M/BD and N = 4 at VB. Average concentrations below the LOQ are shown as ‘<LOQ’. No range is given if all concentrations were <LOQ.

Full name	Abbreviation	Great tit		Blue tit		LOQ
		3M/BD	VB	3M/BD	VB	
Perfluorobutanoic acid	PFBA	<LOQ	<LOQ	<LOQ (<LOQ – 1.13)	<LOQ	0.262
Perfluoropentanoic acid	PFPeA	<LOQ	<LOQ	<LOQ	<LOQ	0.179
Perfluorohexanoic acid	PFHxA	0.431 (<LOQ – 1.25)	<LOQ (<LOQ – 0.440)	<LOQ (<LOQ – 0.782)	<LOQ (<LOQ – 0.763)	0.375
Perfluoroheptanoic acid	PFHpA	<LOQ (<LOQ – 4.03)	<LOQ	<LOQ	<LOQ	0.485
Perfluorooctanoic acid	PFOA	33.3 (0.836 – 243)	1.12 (0.503 – 2.22)	22.6 (1.88 – 64.9)	1.48 (0.505 – 2.21)	0.338
Perfluorononanoic acid	PFNA	7.13 (<LOQ – 26.4)	0.565 (<LOQ – 1.48)	1.65 (0.275 – 3.74)	<LOQ (<LOQ – 0.368)	0.198
Perfluorodecanoic acid	PFDA	7.71 (<LOQ – 26.9)	1.47 (0.641 – 2.67)	2.16 (0.808 – 4.11)	0.993 (0.794 – 1.25)	0.334
Perfluoroundecanoic acid	PFUnDA	8.60 (<LOQ – 53.7)	0.995 (0.344 – 2.17)	1.43 (0.252 – 2.58)	0.420 (0.250 – 0.551)	0.230
Perfluorododecanoic acid	PFDoDA	355 (3.04 – 2953)	8.94 (2.61 – 21.5)	50.6 (4.85 – 91.5)	2.96 (2.13 – 4.43)	0.552
Perfluorotridecanoic acid	PFTrDA	139 (1.19 – 1034)	5.07 (1.46 – 13.9)	18.4 (1.82 – 40.2)	1.21 (0.769 – 2.01)	0.467
Perfluorotetradecanoic acid	PFTeDA	50.1 (<LOQ – 450)	1.77 (<LOQ – 5.10)	5.16 (<LOQ – 15.0)	<LOQ (<LOQ – 1.13)	0.535
Perfluorobutane sulfonate	PFBS	24.9 (<LOQ – 88.5)	4.45 (<LOQ – 35.1)	6.61 (<LOQ – 15.3)	11.5 (<LOQ – 34.5)	0.762
Perfluoropentane sulfonate	PFPeS	1.08 (<LOQ – 16.2)	<LOQ	0.810 (<LOQ – 4.68)	<LOQ	0.671
Perfluorohexane sulfonate	PFHxS	166 (<LOQ – 1005)	<LOQ	116 (<LOQ – 334)	<LOQ	2.79
Perfluoroheptane sulfonate	PFHpS	149 (2.13 – 713)	<LOQ	28.2 (<LOQ – 50.9)	<LOQ	0.569
Perfluorooctane sulfonate	PFOS	15545 (261 – 67916)	101 (34.5 – 253)	3508 (434 – 6483)	34.1 (14.0 – 41.9)	0.506
Perfluorodecane sulfonate	PFDS	1939 (1.61 – 13765)	<LOQ	224 (3.72 – 519)	<LOQ	0.587
4:2 fluorotelomer sulfonate	4:2 FTS	<LOQ	<LOQ	<LOQ	<LOQ	0.857
6:2 fluorotelomer sulfonate	6:2 FTS	<LOQ	<LOQ	<LOQ	<LOQ	1.35
8:2 fluorotelomer sulfonate	8:2 FTS	<LOQ	<LOQ	<LOQ	<LOQ	1.54
Perfluorobutane sulfonamide	FBSA	1.64 (<LOQ – 7.51)	<LOQ	3.68 (<LOQ – 17.9)	<LOQ	0.569
9-chlorohexadecafluoro-3-oxa-nonane-1-sulfonate	9Cl-PF3ONS	<LOQ	<LOQ	<LOQ	<LOQ	0.375
11-chloroeicosafluoro-3-oxaun-decane-1-sulfonate	11Cl-PF3OUdS	<LOQ	<LOQ	<LOQ	<LOQ	0.611
4,8-dioxa-3H-perfluorononanoic acid	NaDONA	<LOQ	<LOQ	<LOQ	<LOQ	0.0982
Hexafluorpropylene oxide-di-mer acid	HFPO-DA	<LOQ	<LOQ	<LOQ	<LOQ	0.173
Perfluoro(2-ethoxyethane) sul-fonate	PFEESA	<LOQ	<LOQ	<LOQ	<LOQ	0.302
Perfluoro-4-oxapentanoic acid	PF4OPeA	<LOQ	<LOQ	<LOQ	<LOQ	0.122

Perfluoro-5-oxahexanoic acid	PF5OHxA	<LOQ	<LOQ	<LOQ	<LOQ	0.361
Perfluoro-3,6-dioxaheptanoic acid	3,6-OPFHpA	<LOQ	<LOQ	<LOQ	<LOQ	0.389

Table S2. PFAS concentrations and limit of quantification (LOQ) in plasma (µg/L) of great tit and blue tit. Values represent averages and range (min – max, between brackets). Sample sites represent the combination of 3M and Blokkersdijk (3M/BD) and Vlietbos (VB). Great tit plasma: N = 14 at 3M/BD, N = 8 at VB. Blue tit plasma: N = 6 at 3M/BD, N = 4 at VB. Average concentrations below the LOQ are shown as '<LOQ'. No range is given if all concentrations were <LOQ.

Full name	Abbreviation	Great tit		Blue tit		LOQ
		3M/BD	VB	3M/BD	VB	
Perfluorobutanoic acid	PFBA	9.39 (<LOQ – 68.5)	<LOQ	24.8 (<LOQ – 149)	<LOQ	3.21
Perfluoropentanoic acid	PFPeA	<LOQ	<LOQ	<LOQ	<LOQ	1.32
Perfluorohexanoic acid	PFHxA	<LOQ	<LOQ	<LOQ	<LOQ	3.12
Perfluoroheptanoic acid	PFHpA	<LOQ	<LOQ	<LOQ	<LOQ	8.75
Perfluorooctanoic acid	PFOA	107 (<LOQ – 389)	1.74 (<LOQ – 13.4)	109 (21.7 – 315)	<LOQ	0.834
Perfluorononanoic acid	PFNA	2.39 (<LOQ – 13.1)	<LOQ	<LOQ	<LOQ	1.04
Perfluorodecanoic acid	PFDA	1.51 (<LOQ – 20.7)	<LOQ	<LOQ	<LOQ	0.896
Perfluoroundecanoic acid	PFUnDA	<LOQ	<LOQ	<LOQ	<LOQ	0.733
Perfluorododecanoic acid	PFDoDA	46.2 (<LOQ – 341)	<LOQ	<LOQ	<LOQ	1.83
Perfluorotridecanoic acid	PFTrDA	11.7 (<LOQ – 94.2)	<LOQ	<LOQ	<LOQ	3.49
Perfluorotetradecanoic acid	PFTeDA	<LOQ	<LOQ	<LOQ	<LOQ	1.38
Perfluorobutane sulfonate	PFBS	5395 (52.1 – 24385)	4588 (41.3 – 12108)	6890 (335 – 19361)	3035 (54.8 – 9411)	6.37
Perfluoropentane sulfonate	PFPeS	<LOQ	<LOQ	<LOQ	<LOQ	8.49
Perfluorohexane sulfonate	PFHxS	<LOQ	<LOQ	<LOQ	<LOQ	15.4
Perfluoroheptane sulfonate	PFHpS	<LOQ	<LOQ	<LOQ	<LOQ	12.2
Perfluorooctane sulfonate	PFOS	4285 (220 – 13821)	<LOQ	5275 (1123 – 14558)	<LOQ	11.6
Perfluorodecane sulfonate	PFDS	<LOQ	<LOQ	<LOQ	<LOQ	7.14
4:2 fluorotelomer sulfonate	4:2 FTS	<LOQ	<LOQ	<LOQ	<LOQ	1.39
6:2 fluorotelomer sulfonate	6:2 FTS	<LOQ	<LOQ	<LOQ	<LOQ	1.48
8:2 fluorotelomer sulfonate	8:2 FTS	<LOQ	<LOQ	<LOQ	<LOQ	1.98
Perfluorobutane sulfonamide	FBSA	<LOQ	<LOQ	<LOQ	<LOQ	10.6
9-chlorohexadecafluoro-3-oxanonane-1-sulfonate	9Cl-PF3ONS	<LOQ	<LOQ	<LOQ	<LOQ	1.79
11-chloroeicosafluoro-3-oxaundecane-1-sulfonate	11Cl-PF3OUdS	<LOQ	<LOQ	<LOQ	<LOQ	1.83
4,8-dioxa-3H-perfluorononanoic acid	NaDONA	<LOQ	<LOQ	<LOQ	<LOQ	0.938
Hexafluorpropylene oxide-dimer acid	HFPO-DA	<LOQ	<LOQ	<LOQ	<LOQ	3.20
Perfluoro(2-ethoxyethane) sulfonate	PFEESA	18.2 (<LOQ – 81.3)	26.2 (<LOQ – 80.0)	28.1 (<LOQ – 77.0)	8.60 (<LOQ – 34.3)	3.60
Perfluoro-4-oxapentanoic acid	PF4OPeA	<LOQ	<LOQ	<LOQ	<LOQ	1.02
Perfluoro-5-oxahexanoic acid	PF5OHxA	<LOQ	<LOQ	<LOQ	<LOQ	1.14
Perfluoro-3,6-dioxaheptanoic acid	3,6-OPFHpA	<LOQ	<LOQ	<LOQ	<LOQ	1.38

Table S3. Mean values and standard error for the different reproductive parameters and nestling body condition at the different locations for both great tit and blue tit.

Reproductive parameter	Great tit		Blue tit	
	3M/BD	VB	3M/BD	VB
Average day 1st egg	14.7 ± 1.51	7.00 ± 1.90	16.2 ± 2.33	5.75 ± 3.20
Shell thickness (µm)	78.2 ± 1.29	91.2 ± 4.56	72.8 ± 1.53	76.3 ± 2.75
Clutch size[a]	8.18 ± 0.464	8.85 ± 0.639	8.67 ± 0.615	9.50 ± 0.645
Hatching success[b]	0.835 ± 0.0821	0.620 ± 0.122	0.967 ± 0.0338	0.977 ± 0.0236
Body condition of nestlings	16.8 ± 0.165	15.4 ± 0.182	11.6 ± 0.128	10.7 ± 0.141
Nestlings'survival[c]	0.933 ± 0.0674	0.827 ± 0.114	1.00	0.917 ± 0.0833

[a]Excluding the egg that was collected for PFAS measurements
[b]Number of hatched eggs divided by the number of incubated eggs, including nests where no eggs hatched
[c]Number of fledglings divided by the number of hatched eggs
